# Biorefining and the Functional Properties of Proteins from Lipid and Pigment Extract Residue of *Chlorella pyrenoidosa*

**DOI:** 10.3390/md17080454

**Published:** 2019-08-01

**Authors:** Kongyong Lu, Xurui Zhao, Shih-Hsin Ho, Ruijuan Ma, Youping Xie, Jianfeng Chen

**Affiliations:** 1 Technical Innovation Service Platform for High Value and High Quality Utilization of Marine Organism, Fuzhou University, Fuzhou 350108, China; 2 Fujian Engineering and Technology Research Center for Comprehensive Utilization of Marine Products Waste, Fuzhou University, Fuzhou 350108, China; 3 Fuzhou Industrial Technology Innovation Center for High Value Utilization of Marine Products, Fuzhou University, Fuzhou 350108, China; 4 State Key Laboratory of Urban Water Resource and Environment, School of Environment, Harbin Institute of Technology, Harbin 150090, China

**Keywords:** microalgal residue, alkaline extraction, proteins, response surface methodology, functional properties

## Abstract

Microalgae are considered as excellent candidates for bioactive compounds, yet microalgal residues remaining after the extraction of one or two compounds are usually discarded, which is not economical. This study demonstrates the alkaline extraction of proteins from *Chlorella pyrenoidosa* residue after lipid and pigment extractions, and their functional properties. Single-factor experiments and response surface methodology were used to obtain the optimal conditions for protein extraction. Based on our results, a maximum protein yield of 722.70 mg/g, was obtained under the following extraction conditions: sodium hydroxide concentration 7.90%, extraction temperature 70.00 °C, extraction time 34.80 min, and microalgal residue concentration 8.20 mg/mL. The molecular weight of microalgal residue protein isolate (MRPI) was mainly distributed at the regions of 0.18–0.50 kDa, 0.50–1.50 kDa, and 1.50–5.00 kDa. The essential amino acid content was greater than the values recommended by FAO/WHO standards; a high essential amino acid index value (1.49) was another good indication that MRPI is suitable for human consumption. Moreover, MRPI exhibited excellent emulsifying properties and antioxidant activity, which suggests it may be useful as an emulsifying agent and antioxidant. These findings could improve the extraction methods of functional protein from microalgal residue and add value to microalgae-based bioactive compound production processes.

## 1. Introduction

Microalgae are photosynthetic microorganisms that are rich in high-value compounds, such as lipids, proteins, carbohydrates, pigments, and vitamins [1,2]. The lipid content of microalgae ranges from 1.5–75% of dry weight, which offers great potential for biodiesel production [3]. Furthermore, many microalgal lipids contain omega-3 unsaturated fatty acids such as eicosapentaenoic acid (EPA) and docosahexaenoic acid (DHA), which can support cardiovascular, brain, and eye systems, prevent cardiovascular disease, and have anti-inflammatory, antibiotic, and anticancer activities in humans [4,5]. On the other hand, microalgae contain large amounts of pigments, including phycobilins, chlorophylls, and carotenoids, to maintain the functions of photosynthetic metabolism [3]. Several carotenoids, such as lutein, astaxanthin, and zeaxanthin, exhibit antioxidant, anticancer, and anti-inflammatory activities [6]. In sum, microalgae are promising sources of lipids and pigments, which can be used as functional foods and pharmaceuticals.

The production of lipids and pigments from microalgae, however, are costly, which limits their economic feasibility [7]. If the microalgal residue is discarded during the lipid and pigment biorefinery process, this renders the process economically inviable; valuable metabolites, such as proteins, remain in the residue. The protein content of *Chlorella pyrenoidosa* residue, for example, could be up to 64.13% after lipid extraction, which could serve as livestock feed to replace soy and fish meals [8]. Also, microalgal residue protein hydrolysates have exhibited good antioxidant and antiaging activity in studies, which indicates their potential for nutraceutical and nutricosmetic applications [9]. Moreover, a polypeptide isolated from *C. pyrenoidosa* has shown antitumor activity, which has the potential for food, nutraceutical, and pharmaceutical applications [10]. Therefore, the utilization of proteins from microalgal residue is of great importance; this study could improve the economic feasibility of microalgae-based lipid and pigment production processes.

Acid-, enzyme-, and alkali-assisted extraction methods have been developed to extract protein from plants. Acid-assisted extraction appears less promising than the other methods, as the protein extraction efficiency is extremely low [11]. Enzyme-assisted extraction has a relatively high extraction efficiency, and does not cause severe corrosion of devices since it is operated at mild pH conditions [12], whereas the costs of this extraction method are much higher than other extraction methods. Alkaline extraction is considered to be the most favorable method for protein extraction, as the process is relatively simple and can be performed with low energy and cost inputs [13]. Moreover, protein solubility can increase in alkaline solution, which improves protein extraction [14]. However, the alkaline extraction process has not been well-optimized, which limits the application of this method for protein extraction from microalgal residue. Response surface methodology (RSM) has been suggested as an effective tool to optimize the process when many factors and their interactions need to be evaluated [15]. 

In this study, the alkaline extraction of proteins from *C. pyrenoidosa* residue was optimized by single-factor experiments and RSM approaches. Furthermore, the molecular weight, amino acid composition, emulsifying properties, and antioxidant activities of MRPI were evaluated for its potential application in functional food and pharmaceutical products. 

## 2. Results and Discussion

### 2.1. Optimization of Protein Extraction Conditions by Single-Factor Experiments

**Figure 1:**
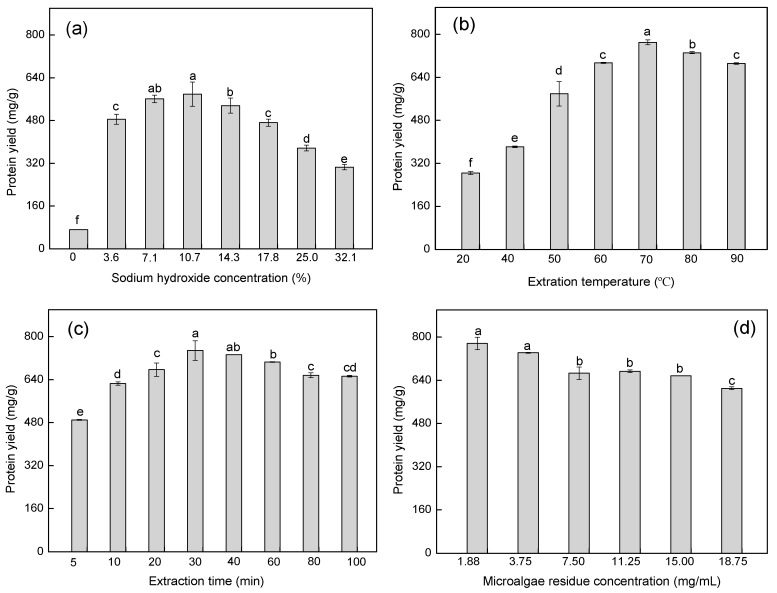
The effect of the single-factor experiments on protein yield. (**a**) Effect of sodium hydroxide concentration on protein yield; (**b**) Effect of extraction temperature on protein yield; (**c**) Effect of extraction time on protein yield; (**d**) Effect of microalgal residue concentration on protein yield. Values are averages of triplicates ± standard deviation. The different lowercase letters represent significant differences at *p* < 0.05.

The effects of sodium hydroxide concentration, extraction temperature, extraction time, and microalgal residue concentration on protein extraction from microalgal residue are shown in Figure 1. The protein yield increased sharply as sodium hydroxide concentration increased from 0.0 to 10.7% (Figure 1a). This could be due to the high alkali concentration, as this promotes the breakdown of hydrogen bonds and the dissociation of hydrogen from proteins, which can enhance protein solubility [16]. However, the protein yield gradually decreased with further increases in sodium hydroxide concentration from 10.7 to 32.1%. This is consistent with a previous study that showed the protein yield from tea residue was enhanced with an increase in alkali concentration, whereas yields decreased under excessively high alkali concentrations [17]. Based on our results, a sodium hydroxide concentration of 7.1–14.3% was chosen for further optimization of protein extraction.

Extraction temperatures exhibited a significant effect on protein extraction (Figure 1b). The protein yield gradually increased when the temperature increased from 20 to 70 °C, whereas yields slightly decreased with further increases in temperature from 70 to 90 °C. The enhanced protein yields associated with rises in temperature could be due to an increase in protein solubility [18]. In addition, the extraction of some cell membrane-associated proteins, such as glycoproteins, are energy-intensive and can be improved at high temperature [16]. Nevertheless, excessively high temperatures are unfavorable for protein extraction due to the occurrence of protein coagulation, denaturation, and hydrolysis [17,19]. Hence, an extraction temperature range of 60–80 °C was chosen for further optimization of protein extraction.

As shown in Figure 1c, protein yield significantly increased with the increase in extraction time from 5 to 30 min, whereas the yield gradually decreased when times were extended further. Some proteins, especially cell wall-associated proteins, are difficult to extract [20], which can take longer times to release. However, a decrease in protein yield under extraction times >30 min could be due to denaturing effects on proteins [21]. Thus, an extraction time range of 20–40 min was chosen for further optimization of protein extraction.

Increases in microalgal residue concentration were associated with negative effects on protein extraction. Protein yield decreased gradually from 776.03 to 666.01 mg/g with an increase in microalgal residue concentration from 1.88 to 7.50 mg/mL, which leveled off from 7.50 to 15.00 mg/mL, and then decreased to 610.10 mg/g at 18.75 mg/mL microalgal residue (Figure 1d). This could be explained by the mass transfer principle, as a higher solvent to sample ratio (lower microalgal residue concentration) had a greater driving force during mass transfer, which enhanced protein extraction [22]. Dried microalgae typically form cell clumps, which could also affect protein extraction if not properly dispersed with a solvent [23]. More cell clumps exist under higher microalgal residue concentrations, which is unsuitable for protein extraction. Moreover, the high viscosity of proteins and polysaccharides under concentrations of high microalgal residue can prevent the separation of proteins during centrifugation [21]. However, excessively low microalgal residue concentrations are also not efficient for protein extraction. Thus, a microalgal residue concentration range of 7.50–15.00 mg/mL was chosen for further optimization of protein extraction.

### 2.2. Optimization of Protein Extraction Conditions by RSM

#### 2.2.1. Model Fitting and Adequacy Checking

As shown in Table 1, the optimization of sodium hydroxide concentration, extraction temperature, extraction time, and microalgal residue concentration were evaluated for their effects on protein yield using the Box–Behnken design, which consisted of four factors, three levels, and five replicates at the central point. The stability and variability of the protein extraction system were evaluated by performing five center-point runs.

The experimental data were analyzed via multiple regression analysis, and the relationship between response variables and test variables was calculated using the following second-order polynomial equation:

*Y* = 675.14 − 33.94*X*_1_ + 6.95*X*_2_ + 19.77*X*_3_ − 39.29*X*_4_ + 6.82*X*_1_*X*_2_ +
7.25*X*_1_*X*_3_ + 19.70*X*_1_*X*_4_ − 41.57*X*_2_*X*_3_ + 0.85*X*_2_*X*_4_ + 6.88*X*_3_*X*_4_ − 25.37*X*_1_^2^ − 27.48*X*_2_^2^ − 25.28*X*_3_^2^ − 6.17*X*_4_^2^

where *Y* is the response variable for protein yield; *X*_1_, *X*_2_, *X*_3_, and *X*_4_ are the test variables for sodium hydroxide concentration, extraction temperature, extraction time, and microalgal residue concentration, respectively.

The ANOVA for this model is shown in Table 2. The *p* value of this model (<0.0001), *p* value of lack of fit (0.0587 > 0.05), determination coefficient (*R*^2^ = 93.23%), and adjusted determination coefficient (Adj. *R*^2^ = 86.46%) indicated that this model had a good fit with the experimental data and theoretical values of protein yield. A relatively low coefficient of variation value (CV = 2.67%) displayed high reproducibility and reliability of experimental values. The linear coefficients of *X*_1_, *X*_3_, and *X*_4_, quadratic term coefficients of *X*_1_^2^, *X*_2_^2^, and *X*_3_^2^, and cross-product coefficients of *X*_1_*X*_4_ and *X*_2_*X*_3,_ were significant (*p* < 0.05); coefficients of the other terms were not significant (*p* ≥ 0.05). The *F* values indicated that the effects of four factors on protein yield were in the order of microalgal residue concentration (*X*_4_) > sodium hydroxide concentration (*X*_1_) > extraction time (*X*_3_) > extraction temperature (*X*_2_); the interaction effects on protein yield were in the order of *X*_2_*X*_3_ > *X*_1_*X*_4_ > *X*_1_*X*_3_ > *X*_3_*X*_4_ > *X*_1_*X*_2_ > *X*_2_*X*_4_.

#### 2.2.2. Response Surface Analysis

**Figure 2:**
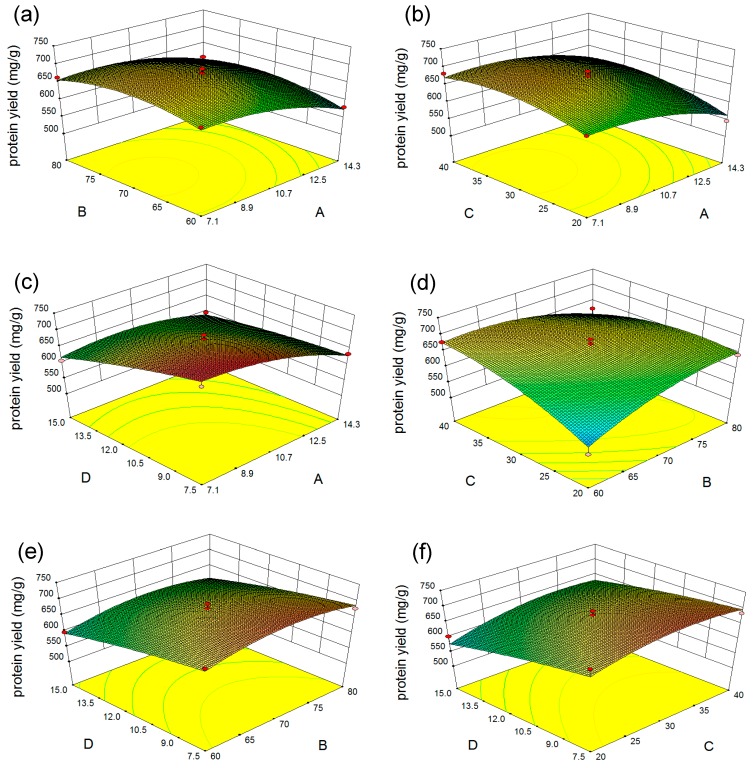
Response surface plots of the interactions between different selected factors. (**a**) Effect of sodium hydroxide concentration and extraction temperature on protein yield; (**b**) Effect of sodium hydroxide concentration and extraction time on protein yield; (**c**) Effect of sodium hydroxide concentration and microalgal residue concentration on protein yield; (**d**) Effect of extraction temperature and extraction time on protein yield; (**e**) Effect of extraction temperature and microalgal residue concentration on protein yield; (**f**) Effect of extraction time and microalgal residue concentration on protein yield. A: sodium hydroxide concentration (%); B: extraction temperature (°C); C: extraction time (min); D: microalgal residue concentration (mg/mL).

To visualize the effects of independent variables on protein extraction, three-dimensional response surface plots of the interactions between different selected factors were established (Figure 2). The response surface slope displays the degree of interaction between the influences of two variables. The results show that the interactions between sodium hydroxide concentration and extraction temperature (Figure 2a), hydroxide concentration and extraction time (Figure 2b), extraction temperature and microalgal residue concentration (Figure 2e), and extraction time and microalgal residue concentration (Figure 2f) on protein yield were not significant. The interactions between extraction temperature and extraction time, however, had a linear effect on protein yield (Figure 2d). Protein yield increased when the extraction temperature increased from 60.00 to 70.13 °C, and the extraction time increased from 20.00 to 34.78 min. Also, the interactive effect of sodium hydroxide concentration and microalgal residue concentration on protein yield was significant (Figure 2c). The protein yield increased with the decrease in microalgal residue concentration from 15.00 to 7.50 mg/mL, and increase in sodium hydroxide concentration from 7.10 to 7.92%.

Based on the response surface model, the predicted optimal conditions for protein extraction were: 7.92% sodium hydroxide; extraction temperature 70.13 °C; extraction time 34.78 min; and 8.17 mg/mL microalgal residue. Under these conditions, the protein yield was predicted to be 724.80 mg/g. For the verification experiment, the extraction conditions were adjusted to the following: 7.90% sodium hydroxide; extraction temperature 70.00 °C; extraction time 34.80 min; and microalgal residue concentration 8.20 mg/mL. Under these conditions, the protein yield of 722.70 ± 3.60 mg/g was obtained, which was not significantly different from the predicted result, indicating that the response surface model was valid.

### 2.3. Molecular Weight Distribution of MRPI

The molecular weight distribution of MRPI is shown in Table 3. It was mainly distributed in the regions of 0.18–0.50 kDa, 0.50–1.50 kDa, and 1.50–5.00 kDa, accounting for 20.81%, 58.99%, and 18.02% of total molecular weight distribution, respectively. This result was different from that of *Tetraselmis* sp., which had molecular weights mainly distributed within the range of 15–50 kDa when extracted under neutral conditions [24]. However, our results were consistent with results for the brown seaweed *Ascophyllum nodosum*, which had an average molecular weight of protein at 2.82 kDa when extracted under alkaline conditions [25]. The low molecular weight of MRPI could be due to alkaline hydrolysis of protein, resulting in short peptides with low molecular weights [25]. On the other hand, physical and mechanical processes, such as ultrasound, have been linked to a decrease in protein molecular weight and protein fragmentation [26]. In our study, MRPI was extracted from the microalgal residue after coextraction of lipid and pigment by ethanol–hexane with bead beating, which may affect protein structure and lead to dissociation of large proteins. The main distribution of small proteins could affect the functional properties of MRPI, such as emulsifying ability and stability as well as antioxidant activity. 

### 2.4. Amino Acid Profile of MRPI

As shown in Table 4, the amino acid composition of MRPI was similar to that of microalgal residue, suggesting that the alkaline extraction did not adversely affect the nutritional value of MRPI. The glutamic acid content was the highest (14.40%), followed by leucine, aspartic acid, alanine, valine, phenylalanine, and arginine. The compositions of cysteine, histidine, and methionine were the lowest, accounting for 0.19%, 0.63%, and 1.65%, respectively. These results are consistent with a previous study, which reported that glutamic acid, leucine, aspartic acid, alanine, valine, phenylalanine, and arginine were major amino acids, whereas cysteine, histidine, and methionine were minor amino acids of the proteins of *Tetraselmis* sp. [24] and *Chlorella vulgaris* [27]. Also, the acidic amino acid content (sum of aspartic acid and glutamic acid) was greater than that of basic amino acids (sum of lysine, arginine, and histidine), which could explain why high protein solubility and enhanced protein extraction were observed in alkaline solution [28]. It is worth noting that all essential amino acids contents were higher than the recommended FAO/WHO standards [29]. Besides, the essential amino acid index (EAAI) value of MRPI reached 1.49, which is higher than that of soybean protein [27] and similar to that of microalgal residue (1.43) and *Chlorella vulgaris* protein [27]. An EAAI greater than 1 indicates good quality for human consumption [30,31]. Therefore, MRPI could be applied as a nutritional food.

### 2.5. Emulsifying Ability and Stability of MRPI

The emulsifying activity (EA) and emulsifying stability (ES) values for MRPI are shown in Table 5. Results indicate that the EA of MRPI is similar to that of commercial emulsifying agent (soy protein isolate and Na-caseinates), and better than that of whey protein and egg white protein. It was reported that EA is affected by molar mass, charge, and physicochemical factors such as pH, ionic strength, and temperature [32]. Thus, the main distribution of small proteins could be one reason for the high EA value of MRPI. Also, an alkaline pH could increase EA value by altering the charge of protein molecules [33]. 

No significant difference of ES was observed between MRPI and soy protein isolate along with Na-caseinates; however, the ES of MRPI was greater than that of whey protein and egg white protein. The high ES of MRPI could be attributed to the high surface hydrophobicity, as hydrophobic surface interactions on proteins can form a strong oil–water interface and result in a stable emulsion [34]. Emulsified protein accounted for 84% of MRPI, which was similar to soy protein isolate, and higher than Na-caseinates, whey protein and egg white protein. Therefore, MRPI with high EA, ES, and emulsified protein content, could be used as an emulsifying agent.

### 2.6. Antioxidant Activity of MRPI

**Figure 3:**
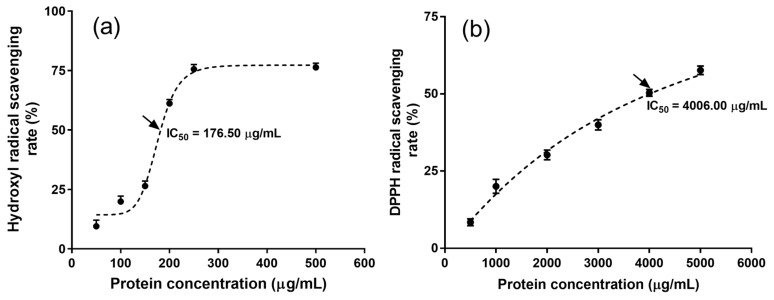
Antioxidant activity of MRPI. (**a**) Hydroxyl free radical scavenging rate; (**b**) DPPH free radical scavenging rate. Values are averages of triplicates ± standard deviation.

The antioxidant activity of MRPI was investigated to further evaluate the applicable value of MRPI. Hydroxyl radicals are deemed an extremely reactive free radical in living organisms, which easily react with biological molecules (e.g., amino acids, proteins, and DNA) and lead to cell damage [35,36]. The hydroxyl radical scavenging rate of MRPI slightly enhanced as protein concentration increased from 50 to 150 μg/mL, followed by a sharp increase with a rise in protein concentration from 150 to 250 μg/mL, and a plateau from 250 to 500 μg/mL protein (Figure 3a). The IC_50_ (176.50 μg/mL) of the hydroxyl radical scavenging rate was significantly greater than that of many reported proteins, such as those from sweet potato [37] and rapeseed [38].

The 2,2-diphenyl-picrylhydrazyl (DPPH) radicals are considered stable and have been used widely to evaluate free radical scavenging activity [35]. The DPPH radical scavenging rate of MRPI gradually increased as protein concentration increased from 500 to 5000 μg/mL (Figure 3b). The IC_50_ of the DPPH radical scavenging rate was 4006.00 μg/mL, which was much lower than that of the hydroxyl radical scavenging rate. Nevertheless, the IC_50_ of the DPPH radical scavenging rate was similar to the values reported for many proteins, such as *Octopus vulgaris* protein [39] and goby muscle protein [40]. Therefore, the MRPI obtained by alkaline extraction exhibited good antioxidant activity, and could be used as a natural antioxidant source.

## 3. Materials and Methods

### 3.1. Microalgal Residue

The dried powder of *C. pyrenoidosa*, a saline water tolerant strain, was purchased from Fuqing King Dnarmsa Spirulina Co., Ltd, Fujian, China. Lipid and pigment were coextracted from *C. pyrenoidosa* biomass by a method using ethanol-hexane (3:1, v/v), as previously reported [41]. Microalgal residue was collected by centrifugation at 8800× *g* for 5 min, freeze-dried for 24 h, and stored at −80 °C.

### 3.2. Optimization of Protein Extraction Conditions

To investigate the optimal parameters for protein extraction from the microalgal residue, sodium hydroxide concentration, extraction temperature, extraction time, and microalgal residue concentrations were evaluated. To test the effects of sodium hydroxide concentration (0, 3.6, 7.1, 10.7, 14.3, 17.8, 25.0, and 32.1%), conditions were set as follows: extraction temperature 50 °C; extraction time 30 min; and microalgal residue concentration 3.75 mg/mL. For extraction temperature (20, 40, 50, 60, 70, 80, and 90 °C), conditions were: sodium hydroxide concentration 10.7%; extraction time 30 min; and microalgal residue concentration 3.75 mg/mL. For extraction time (5, 10, 20, 30, 40, 60, 80, and 100 min), conditions were: sodium hydroxide concentration 10.7%; extraction temperature 70 °C; and microalgal residue concentration 3.75 mg/mL. For microalgal residue concentration (1.88, 3.75, 7.50, 11.25, 15.00, and 18.75 mg/mL), conditions were: sodium hydroxide concentration 10.7%; extraction temperature 70 °C; and extraction time 30 min. Extracts were centrifuged at 8800× *g* for 2 min, then the supernatant was collected to assess protein concentration.

Based on a three-level four-factor Box–Behnken design (BBD), RSM was performed to evaluate the optimal conditions of protein extraction. Sodium hydroxide concentration (*X*_1_), extraction temperature (*X*_2_), extraction time (*X*_3_), and microalgal residue concentration (*X*_4_) were investigated as independent variables. The average of independent parameters obtained from three experiments were fitted to a second order polynomial model as follows:

$$Y=\beta_{o}+\sum_{i=1}^{k}\beta_{i}X_{i}+\sum_{i=1}^{k}\beta_{ii}X_{i}^{2}+\sum_{i=1}^{k-1}\sum_{j>1}^{k}\beta_{ij}X_{i}X_{j}$$

where *Y* is the response variable, *X_i_* and *X_j_* are independent variables, *k* is the number of tested variables (*k* = 4), and *β_o_*, *β_i_*, *β_ii_*, *β_ij_* were intercept, linear, quadratic, and cross-product coefficients, respectively. 

Analysis of variance (ANOVA) was carried out to determine the individual linear, quadratic, and interaction regression coefficients (*β*) using Design Expert 8.0.6 software (Stat-Ease, Inc., Minneapolis, MN, USA). The coefficient of determination (*R*^2^) was used to assess the fitness of the polynomial equation to the responses, and the significance of the dependent variables was statistically analyzed by calculating the *p* value.

### 3.3. Determination of Protein Yield

Protein concentration was determined using the biuret method. Briefly, 1.0 mL protein solution was mixed with 4.0 mL of biuret reagent and then kept at room temperature for 30 min. The optical density at 540 nm (OD_540_) was measured using an ultraviolet-visible spectrophotometer (model U-2001, Hitachi, Tokyo, Japan). Bovine serum albumin (BSA) was used as a protein standard. The protein concentration was determined by the calibration curve:

$$y=19.256x-0.106\;(R^{2}=0.999)$$

where *y* and *x* represent the protein concentration (mg/mL) and OD_540_, respectively.

The protein yield was calculated using the following formula:

$$Y=\frac{C\times V}{M}$$

where *Y* is protein yield (mg/g), *C* is protein concentration (mg/mL), *V* is volume of the protein extract (8 mL), *M* is the weight of microalgal residue (g).

### 3.4. Purification of MRPI

Protein extract was thoroughly mixed with ethanol at a ratio of 1:3 (v/v), centrifuged at 8800× *g* for 2 min, and the supernatant was collected. Ethanol was then removed in a rotary evaporator under reduced pressure and the pH was adjusted to 7.0 with hydrochloric acid. Protein samples were desalted by dialysis at 4 °C for 72 h, followed by lyophilization for 24 h. The obtained MRPI was analyzed for molecular weight distribution, amino acid composition, emulsifying properties, and antioxidant activity.

### 3.5. Determination of the Molecular Weight of MRPI

In short, 20.0 mg MRPI was dissolved in 10 mL solvent (acetonitrile/water/trifluoroacetic acid, 45:54.9:0.1, v:v:v), and then filtered through a 0.22 μm polytetrafluoroethylene filter. The molecular weight distribution of MRPI was determined using an Agilent 1200 high-performance liquid chromatography instrument (Agilent, Santa Clara, CA, USA). Samples (10 μL) were injected into a TSKgel 3000 SW column (7.8 nm × 30 cm × 10 μm, Merck, Darmstadt, Germany) controlled at 30 °C with a flow rate of 0.5 mL min^-1^. The mobile phase was acetonitrile/water/trifluoroacetic acid (45:54.9:0.1, v:v:v). Protein was detected at a wavelength of 220 nm. The protein standards (Sigma-Aldrich, St. Louis, MO, USA) cytochrome C (12.5 kDa), aprotinin (6.5 kDa), bacitracin (1.45 kDa), ethoxy-alanine-tyrosine-arginine (0.451 kDa), and hippuric acid (0.179 kDa) were used for quantification.

### 3.6. Determination of the Amino Acid Composition of MRPI

In short, 10 mg MRPI was hydrolyzed in 10 mL 6 M HCl at 110 °C for 22 h under nitrogen gas. Distilled water was added to the hydrolysate to adjust the volume to 50 mL. One milliliter of hydrolysate was then dried using a vacuum desiccator at 45 °C and redissolved in 2 mL water. This step was repeated twice. Ultimately, the hydrolyzed protein was dissolved in 1 mL sodium citrate buffer (pH 2.2), which was used for the determination of amino acid composition.

The identification and quantification of amino acids were carried out using an automatic amino acid analyzer (Biochrom 30+ series, Biochrom, Cambridge, UK) with an amino acid mixture (Sigma-Aldrich, St. Louis, MO, USA) as a standard. The amino acid content was calculated using the following formula:

$$X=\frac{c\times f\times V\times M}{m\times 10^{9}}\times 100$$

where *X* is the amino acid content (g/100 g protein), *c* is the amino acid concentration (nmol/μL), *f* is sample dilution factor, *V* is volume of hydrolyzed sample (mL), *M* is molecular weight of the amino acid, and *m* is the weight of sample (g). 

The EAAI was calculated according to a previous study [30] using the following formula:

$$EAAI=\sqrt[{n}]{\frac{aa1}{AA1}\times \frac{aa2}{AA2}\times \cdots \times \frac{aan}{AAn}}$$

where *aan* and *AAn* are the essential amino acid content over total protein content (mg/g) in the sample and FAO/WHO reference [29], respectively. 

### 3.7. Determination of the Emulsifying Properties of MRPI

The emulsifying activity and stability of protein were determined as described previously [42]. The MRPI, soy protein isolate, Na-caseinates, whey protein, and egg white protein were dissolved in reverses osmosis (RO) water at pH 12 and then adjusted to pH 7. Each experiment was carried out using biological triplicates. 

Emulsifying activity (EA) was calculated using the following formula:

$$EA_{}(\%)=\frac{height\;of\;emulsified\;layer}{total\;height\;of\;mixture}\times 100$$

Emulsifying stability (ES) was calculated using the following formula:

$$ES_{}(\%)=\frac{height\;of\;the\;remaining\;layer}{total\;height\;of\;mixture}\times 100$$

The samples in the bottom (aqueous) layer were collected and centrifuged at 8800× *g* for 5 min to remove oil droplets. The oil-free aqueous layer was used to determine the unemulsified protein. The emulsified protein was determined by subtracting unemulsified protein from total protein.

### 3.8. Determination of the Antioxidant Activites of MRPI

The hydroxyl radical scavenging rate was determined according to a previous method [43] with modification. In brief, 3 mL FeSO_4_ (2 mM), 3 mL H_2_O_2_ (1 mM), and 3 mL salicylic acid (6 mM) were thoroughly mixed and incubated at 37 °C for 15 min. The mixture was added with 1 mL various sample concentrations (0, 50, 100, 150, 200, 250, and 500 μg/mL), and incubated at 37 °C for another 15 min. In this reaction, hydroxyl radicals were generated by the Fenton reaction (Fe^2+^ + H_2_O_2_), and salicylic acid was converted into dihydroxy-benzoic acid, which has an absorbance at the wavelength of 510 nm. The hydroxyl radical scavenging rate was calculated using the following equation:

$$Hydroxyl\;radical\;scavenging\;rate\;(\%)=\frac{A0-A1-A2}{A0}\times 100$$

where *A_0_* is the absorbance of the control sample (reaction solution without sample); *A_1_* is the absorbance of the test sample (reaction solution with sample); *A_2_* is used to eliminate the interference of volume difference between *A_0_* and *A_1_*, which is calculated by subtracting *A_0_* from the absorbance of *A_1_* with a 0 μg/mL sample.

The determination of 1,1-diphenyl-2-picrylhydrazyl (DPPH) radical scavenging rate was according to previous studies [43,44] with modification. In brief, 2 mL DPPH (20 mM in absolute ethanol) was mixed with 2 mL of various sample concentrations (0, 500, 1000, 2000, 3000, 4000, and 5000 μg/mL), and incubated at room temperature for 30 min. The mixture was measured at a wavelength of 517 nm. The DPPH radical scavenging rate was determined by the following equation:

$$DPPH\;scavenging\;rate\;(\%)=(1-\frac{A1-A2}{A0})\times 100$$

where *A*_0_ is the absorbance of the control sample (reaction solution of 2 mL DPPH and 2 mL absolute ethanol); *A*_1_ is the absorbance of the test sample (reaction solution of 2 mL DPPH and 2 mL sample); *A*_2_ is the absorbance of the blank (reaction solution of 2 mL sample and 2 mL absolute ethanol). IC_50_ values of hydroxyl and DPPH radical scavenging rate were calculated using Prism 6 (GraphPad Software Inc., San Diego, CA, USA) with dose–response simulation of nonlinear regression.

### 3.9. Statistical Analysis

The experiments of protein extraction and functional properties analysis were performed in triplicate. The statistical differences between groups were determined by one-way ANOVA analysis using IBM SPSS Statistics 18 (Chicago, IL, USA), followed by Duncan’s test with a significant level of 0.05.

## 4. Conclusions

In this study, proteins were extracted from lipid and pigment extract residue by alkaline extraction. The maximum protein yield of 722.70 was obtained under the optimized extraction conditions of sodium hydroxide concentration 7.90%, extraction temperature 70.00 °C, extraction time 34.80 min, and microalgal residue concentration 8.20 mg/mL. The MRPI were mainly small proteins with molecular weights less than 5.00 kD. The extraction procedure did not adversely affect amino acid composition, and the high essential amino acid content and great EAAI indicated that MRPI could be applied as a nutritional food. Moreover, MRPI exhibited excellent emulsifying properties and antioxidant activity in the present study, indicating its potential application as an emulsifying agent and antioxidant. This study will help to provide an efficient extraction method for functional proteins from microalgal residues and could add value to lipid and pigment production.

## Figures and Tables

**Table 1:** Response surface design and the response values for protein yield (mg/g).

Runs	Sodium Hydroxide Concentration (%) (*X*_1_)	Extraction Temperature (°C) (*X*_2_)	Extraction Time (min) (*X*_3_)	Microalgal Residue Concentration (mg/mL) (*X*_4_)	Protein Yield (mg/g) (*Y*)
1	−1(7.1)	−1(60)	0(30)	0(11.25)	661.99
2	1(14.3)	−1(60)	0(30)	0(11.25)	580.09
3	−1(7.1)	1(80)	0(30)	0(11.25)	663.72
4	1(14.3)	1(80)	0(30)	0(11.25)	609.07
5	0(10.7)	0(70)	−1(20)	−1(7.50)	693.98
6	0(10.7)	0(70)	1(40)	−1(7.50)	683.62
7	0(10.7)	0(70)	−1(20)	1(15.00)	602.86
8	0(10.7)	0(70)	1(40)	1(15.00)	620.02
9	−1(7.1)	0(70)	0(30)	−1(7.50)	722.48
10	1(14.3)	0(70)	0(30)	−1(7.50)	633.35
11	−1(7.1)	0(70)	0(30)	1(15.00)	607.84
12	1(14.3)	0(70)	0(30)	1(15.00)	597.47
13	0(10.7)	−1(60)	−1(20)	0(11.25)	530.00
14	0(10.7)	1(80)	−1(20)	0(11.25)	642.97
15	0(10.7)	−1(60)	1(40)	0(11.25)	678.30
16	0(10.7)	1(80)	1(40)	0(11.25)	624.99.
17	−1(7.1)	0(70)	−1(20)	0(11.25)	646.43
18	1(14.3)	0(70)	−1(20)	0(11.25)	546.15
19	−1(7.1)	0(70)	1(40)	0(11.25)	681.98
20	1(14.3)	0(70)	1(40)	0(11.25)	610.80
21	0(10.7)	−1(60)	0(30)	−1(7.50)	682.49
22	0(10.7)	1(80)	0(30)	−1(7.50)	677.32
23	0(10.7)	−1(60)	0(30)	1(15.00)	597.66
24	0(10.7)	1(80)	0(30)	1(15.00)	595.86
25	0(10.7)	0(70)	0(30)	0(11.25)	688.89
26	0(10.7)	0(70)	0(30)	0(11.25)	675.59
27	0(10.7)	0(70)	0(30)	0(11.25)	667.18
28	0(10.7)	0(70)	0(30)	0(11.25)	669.89
29	0(10.7)	0(70)	0(30)	0(11.25)	674.09

**Table 2:** Analysis of variance (ANOVA) of regression parameters ^a,b^.

Source	Degrees of Freedom	Sum of Squares	Mean Square	*F*-Value	*p*-Value
Model	14	56,420.89	4030.06	13.78	<0.0001 **
*X_1_*	1	13,824.44	13,824.44	47.25	<0.0001 **
*X_2_*	1	579.63	579.63	1.98	0.1811
*X_3_*	1	4688.65	4688.65	16.03	0.0013 **
*X_4_*	1	18,526.02	18,526.02	63.32	<0.0001 **
*X_1_X_2_*	1	186.32	186.32	0.64	0.4382
*X_1_X_3_*	1	210.25	210.25	0.72	0.4108
*X_1_X_4_*	1	1552.36	1552.36	5.31	0.0371 *
*X_2_X_3_*	1	6913.92	6913.92	23.63	0.0003 **
*X_2_X_4_*	1	2.89	2.89	0.01	0.9222
*X_3_X_4_*	1	189.06	189.06	0.65	0.4349
*X_1_* ^2^	1	4174.94	4174.94	14.27	0.0020 **
*X_2_* ^2^	1	4899.16	4899.16	16.75	0.0011 **
*X_3_* ^2^	1	4146.19	4146.19	14.17	0.0021 **
*X_4_* ^2^	1	246.93	246.93	0.84	0.3738
Residual	14	4095.80	292.56	-	-
Lack of Fit	10	3814.67	381.47	5.43	0.0587
Pure Error	4	281.13	70.28	-	-
Total	28	60,516.69	-	-	-
R^2^	93.23%				
Adj R^2^	86.46%				
CV	2.67%				

^a,^* Significant at 0.01 < *p* < 0.05, ** significant at *p* < 0.01. ^b^ Source: ANOVA using Design-Expert 8.0.6.

**Table 3:** Molecular weight distribution of microalgal residue protein isolate (MRPI).

Molecular Weight Distribution	Content (%)
>10.00 kDa	1.45
5.00–10.00 kDa	0.18
1.50–5.00 kDa	18.02
0.50–1.50 kDa	58.99
0.18–0.50 kDa	20.81
<0.18 kDa	0.55

**Table 4:** Amino acid composition of different proteins (g/100 g protein).

Amino Acid Composition	MR	MRPI
Threonine *	4.82	3.51
Valine *	5.68	6.93
Methionine *	1.59	1.65
Isoleucine *	4.09	4.69
Leucine *	9.19	10.99
Phenylalanine *	5.80	6.57
Lysine ^b^ *	5.50	5.28
Histidine ^b^	1.77	0.63
Arginine ^b^	5.62	5.85
Aspartic acid ^a^	9.10	10.47
Serine	4.22	2.57
Glutamic acid ^a^	11.63	14.40
Proline	4.69	5.00
Glycine	5.66	4.53
Alanine	7.69	7.94
Cystine	0.44	0.19
Tyrosine	3.53	3.65
EAAI ^c^	1.43	1.49

^a^ Acidic amino acids; ^b^ basic amino acids; ^c^ the calculation of EAAI did not include tryptophan; * essential amino acids. MR: microalgal residue; MRPI: microalgal residue protein isolate.

**Table 5:** Emulsifying ability (EA), emulsion stability (ES), and emulsified protein content for different protein samples.

Protein Types	EA (%)	ES (%)	Emulsified Protein Content (%)
MRPI	57 ± 1.2	55 ± 1.6	84 ± 2.7
Soy protein isolate	59 ± 1.8	58 ± 2.9	89 ± 3.2
Na-caseinates	56 ± 2.1	53 ± 3.1	72 ± 1.2
Whey protein	48 ± 0.9	48 ± 1.5	78 ± 2.5
Egg white protein	52 ± 2.3	48 ± 0.8	68 ± 1.9

Values are averages of triplicates ± standard deviation.
